# Role of Serum Osteoprotegerin as a Diagnostic Indicator of Primary Osteoporosis in Perimenopausal and Postmenopausal Women: An Indian Perspective

**DOI:** 10.5704/MOJ.1803.006

**Published:** 2018-03

**Authors:** A Pandey, YA Khan, SS Kushwaha, F Mohammed, A Verma

**Affiliations:** Department of Orthopaedics, ERA’s Lucknow Medical College and Hospital, Lucknow, India

**Keywords:** osteoporosis, osteoprotegerin, biomarkers

## Abstract

**Introduction:** Osteoporosis (OP) is a major health problem in the older population. The aim of the study was to assess the role of serum osteoprotegerin (OPG) as a diagnostic indicator of primary osteoporosis in peri- and post-menopausal women in an Indian population.

**Materials and Methods:** After ethical approval, 90 cases (45 cases and 45 controls) of peri- and post-menopausal females above 40 years of age presenting to our outpatient department were included in the study. It was a case controlled study conducted between July 2014 to July 2015. Based on the clinical features, t-score and radiological evidence from the DEXA scan, they were equally divided into two groups (cases and controls). Serum osteoprotegerin (OPG) levels were measured amongst cases and controls.

**Results:** The total calcium (mg/dl) level was lower among the cases and the difference was significant (p-Value= <0.001). Similarly, alkaline phosphatase (u/l), osteoprotegerin (u/ml) levels were higher in the cases as compared to controls and the difference was significant (p-Value= <0.001). The mean osteoprotegerin level showed a slight increase with increase in severity of the grading of BMD of spine. The results suggested a cut-off value of ≥10.5 u/ml (86.7% sensitive and 80% specific with accuracy of 84.5%) between normal and osteoporosis.

**Conclusion:** From the present study, we conclude that osteoprotegerin is a valid biomarker to diagnose postmenopausal women with low bone mineral density.

## Introduction

Osteoporosis (OP) is a major health problem among the older adults, particularly in older women. It affects millions of people throughout the world and its frequency increases with age. Osteoporosis is characterized by an abnormally low bone mass and defect in bone architecture leading to increased bone fragility and chances of fracture^1^. At the cellular level, the bone tissues in the adults undergo a continuous process of remodelling in which the bone resorbing cells (osteoclasts) remove the old bone and bone forming cells (osteoblasts) replace the old bone with newly synthesized bone^2^. When the resorption is more than the formation, bone density is reduced and micro-architecture is disturbed leading to osteoporosis and increased bone fragility and chances of fracture^3^. The most common method employed to diagnose and categorize osteoporosis is bone mineral density (BMD) in different locations. World Health Organization (WHO) defines osteoporosis as bone mineral density (BMD) measured by dual energy radiograph absorptiometry (DEXA) scan less than -2.5 standard deviation below the mean value for young adults for same age and sex (T-score). Based on bone mineral density (BMD) osteoporosis is further classified as osteopenia, osteoporosis and severe osteoporosis^4^. Till now the bone strength prediction and fracture risk are mainly based on densitometric measurements. Recently various bone turns over markers have been identified to assess bone turnover rate which can also be used to monitor osteoporosis treatment.

In the year 1997, a few research groups identified a protein, named osteoprotegerin (OPG)^5-7^. Protein osteoprotegerin belongs to the tumour necrosis factor receptor (TNFR) family and is produced by osteoblasts. It is also produced by other cells like peripheral blood lymphocytes^8-10^. Osteoprotegerin acts as a soluble decoy for the receptor activator for nuclear factor K B Ligand (RANKL)^6^. It has already been proved that osteoprotegerin inhibits apoptosis by binding to the tumour necrosis factor (TNF) associated ligand (TRAIL, tumour necrosis factor related apoptosis inducing ligand)^11^. Many recent studies have proved the importance of the osteoprotegerin / RANK / RANKL system in the development of bone diseases^12^. The relationship between bone turnover markers (BTM) and the osteoprotegerin / RANK / RANKL system has not been fully established yet. Several studies demonstrated the direct and inverse correlation between the osteoprotegerin / RANKL and bone mineral density (BMD)^13,14^.

We believe that there is no study done on the role of osteoprotegerin on primary osteoporosis in peri- and post-menopausal women in India. The aim of the study was to assess the role of serum osteoprotegerin (OPG) as a diagnostic indicator of primary osteoporosis in peri- and post-menopausal women in an Indian population in Lucknow, Uttar Pradesh, India.

## Materials and Methods

The study was conducted in the Department of Orthopaedics and Biochemistry in ERA’s Lucknow Medical College and Hospital, Lucknow. It was a case controlled study conducted between July 2014 and July 2015. After ethical approval from the institutional ethics committee, 90 patients were recruited into the study. The study patients included 90 peri-and post-menopausal women. Based on the clinical features, t-score and radiological evidence from the DEXA scan, they were divided into two groups, Cases (n = 45) and Controls (n = 45). Females above 40 years of age with t-score below -1 SD were selected as cases and females with t-score above -1 SD were selected as controls. Females below 40 years of age, pregnant women, female with secondary osteoporosis, bone tuberculosis, liver disorders, alcoholism, thyroid and parathyroid disorders, women on medication with corticosteroids and heparin were excluded from the study.

Patients were recruited from the outpatient clinic of the Department of Orthopaedics. History taking and thorough physical examination and relevant investigations wherever required were done to exclude secondary osteoporosis. The recruited patients were informed of the purpose and relevance of the study. Those who agreed were included in the study after informed and written consent. All the patients were subjected to DEXA (three point) scan. Those whose t-score was more than -1 SD were taken into the control group and the others into cases. There were 45 cases and 45 controls. Observing aseptic precautions, 5ml whole venous blood sample of the recruited cases and controls was drawn and centrifuged. The serum was separated and stored in small capped vials for long term use at -20°C until tested. Serum calcium, phosphorus and alkaline phosphatase levels were obtained. Serum osteoprotegerin level was measured by the ELISA kit, following instructions in the technical bulletin supplied along with the kit. After obtaining the result, serum osteoprotegerin levels were compared with the BMD and the relationship was assessed.

Proximal femur (neck, trochanter) and total hip regions and lumbar spine (L2–L4 region) BMD measurements (g/cm^2^) were obtained by dual energy radiograph absorptiometry (DEXA) with the use of a lunar DPX (GE medical system). The interpretation of BMD was done as t-score according to WHO criteria: t-score of 1.0 as normal, t-score between -1.0 to -2.5 as osteopenia and t-score <-2.5 osteoporosis.

Serum osteoprotegerin was estimated by using the ELH-OPG-1 human-OPG-ELISA kit [RayBiotech Inc., Norcross, USA] according to the manufacturer’s instructions. 100 μl standard or sample was added to each well and incubated for 2.5 hours at room temperature or overnight at 40°C. After that 100 μl prepared biotin antibody was added to each well. After one hour incubation at room temperature, 100 μl streptavidin solution was added. After 45 minute incubation and the last washing step, the remaining conjugate was allowed to react with the substrate H_2_O_2_-tetramethylbenzidine (TMB). 100 μl TMB one-step substrate reagent was added to each well. The reaction was stopped by addition of acidic solution and absorbance of the resulting yellow product was measured at 450 nm.

Data was collected, revised, verified, edited and analysed statistically using SPSS statistical package (version 20.0). Descriptive statistics of all variables were presented as percentage; mean±SD. Statistical analysis was performed by describing the demographic characteristics of study participants. T-tests were used to test differences in the distribution of continuous variables, and the Chi-square test was used to test for differences in the distribution of categorical variables. Diagnostic validity of osteoprotegrin in osteoporosis was observed by receiver operating characteristic curve (ROC curve).

## Results

Comparative demographics as well as clinical parameters of the members of both cases as well as control groups are shown in Table I. On perusal of the demographic parameters (age, weight, height, BMI,) of the members of both the groups, we found no significant difference between the members of the two groups which made the two groups comparable. Mean age since last menstruation was higher among the cases and the difference was significant. As regards to the clinical parameters, the total calcium (mg/dl) level was lower among the cases and the difference was significant (p-Value=<0.001). Similarly, alkaline phosphatase (u/l), and osteoprotegrin (u/ml) levels were higher in the cases as compared to controls and the difference was significant (p-Value = <0.001) (Table I).

**Table I:** Shows distribution of different parameters in cases and control group

	Control (n=45) (p-Value)	Cases (n=45) Mean±SD	Significance Mean±SD
Age (year)*	57.40±9.57	57.58±9.35	0.930
Age since last menstruation (year)*	4.34±2.8	9.95±5.4	<0.001
Weight (kg)*	60.47±16.22	65.22±17.19	0.181
Height (cm)*	154.07±7.44	156.22±8.25	0.197
BMI*	25.04±4.53	26.31±4.59	0.192
Marital status			
Married#	35 (77.78%)	39 (86.67%)	
Unmarried#	4 (8.89%)	1 (2.22%)	0.35
Widow#	6 (13.33%)	5 (11.11%)	
Total calcium (mg/dl)*	9.64±0.49	8.3±1.91	<0.001
T-score			
Lumbar spine*	1.20±0.83	-3.20±1.29	<0.001
Femoral neck*	-0.56±0.50	-1.38±0.98	<0.001
Total hip*	1.56±1.09	-1.22±0.73	<0.001
Alkaline phosphatase (u/l)*	57.93±5.12	79.00±4.08	<0.001
Osteoprotegerin (u/ml)*	9.17±2.91	13.49±2.89	<0.001

* = value express in mean±standard deviation

# = value express in frequency (%)

The mean of osteoprotegerin level in the 45 controls was 9.17±2.91 u/ml. Among cases group, there were 15 (33.3%) patients with low bone mass, eight (17.8%) with mild, 17 (37.8%) with moderate and five (11.1%) with severe osteoporosis. The mean osteoprotegerin level showed a slight increase with increase in severity of grading of BMD of spine. [χ^2^= 38.223; p<0.001] (Table II).

**Table II:** Shows the distribution of osteoprotegerin (u/ml) level between BMD grading

BMD grading of spine							
Osteo-protegerin (u/ml)	Normal* (n=45)	Low bone bone mass (osteopenia)* (n=15)	Mild osteoporosis* (n=8)	Moderate Osteoporosis* (n=17)	Severe osteoporosis (n=5)	F-value	p-Value
Mean (SD)	9.17(2.91)	11.20(2.09)	12.50(1.90)	13.80(2.11)	14.20(2.2)	13.67	<0.001

* = a normal value (the t score was more than -1), osteopenia (the t score was less than -1 and more than -2.5), mild (t score -2.5 through -3), moderate (t score -3.1 through -4), or severe (t score <or=-4.1)

**Fig. 1:**
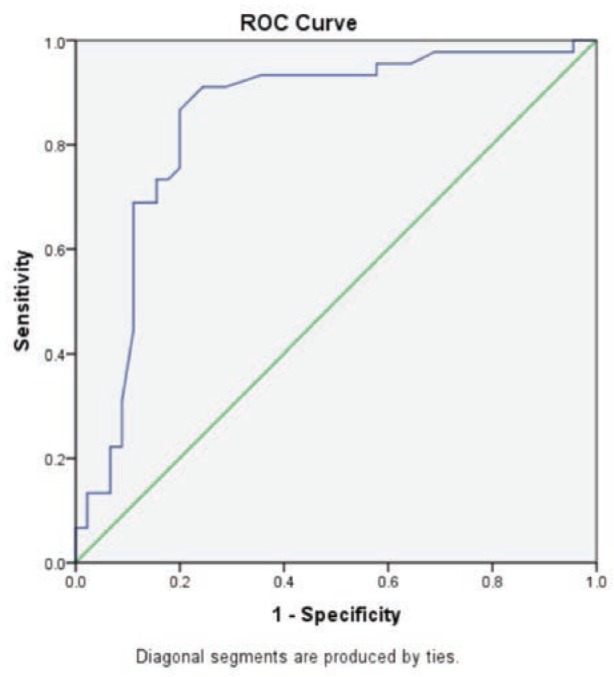
Receiver Operator Characteristic (ROC) curve of osteoprotegerin of cases.

Receiver operator curve (ROC) analysis was done based on the direction of assessment. Osteoprotegerin level was evaluated for prediction of cut-off values between control group and cases group. The results suggested a cut-off value of ≥10.5 u/ml (86.7% sensitive and 80% specific with accuracy of 84.5%) between control group and cases group (Fig. 1).

The results of the Pearson’s correlation test showed a significant inverse correlation between the serum osteoprotegerin (u/ml) concentration and BMD i.e. The total hip (r = -0.568, p<0.01), lumbar spine (r = -0.588, p<0.001), femoral neck (r = -0.347, p<0.05) and total calcium (r = - 0.331,p<0.01) (Table III).

**Table III:** Receiver Operator Characteristic (ROC) curve of osteoprotegerin (u/ml) of cases

Area under the curve				
Test result variable(s): osteoprotegerin (u/ml)				
Area	Std. Error^a^	Asymptotic sig.^b^	Asymptotic 95% confidence interval	
			Lower bound	Lower bound
.845	.045	.000	.757	.933
The test result variable(s): osteoprotegerin (u/ml) has at least one tie between the positive actual state group and the negative actual state group.				

A Under the nonparametric assumption

B Null hypothesis: true area = 0.5

## Discussion

Osteoporosis is a major worldwide public health problem which causes significant morbidity, mortality, and socioeconomic burden^15^. It is defined as a disorder of skeletal system which leads to high risk of fragility fractures due to low bone strength. Maximum bony weakness occurs in peri-or post-menopausal women and is associated with insufficiency of oestrogen, a menopausal condition^16^.

Higher circulating osteoprotegerin levels are often found in patients with osteoporosis and are usually regarded as a reflection of the increased bone turnover and a compensatory response to excessive osteoclast activity^17-19^. Hence in the present study, serum osteoprotegerin was significantly increased in cases (osteoporotic patients) than the control patients and showed a significant inverse correlation between serum osteoprotegerin concentration and bone mineral density of hip, lumbar spine and femoral neck.

Similarly, Youssef *et al* expressed results of osteoprotegerin as mean±SD using students t-test. Serum osteoprotegerin was significantly increased in reduced bone mineral density group (P ≤0.001) when compared to the normal bone mineral group^20^. This was also supported by the work of Rogers *et al*, who stated that a significant negative correlation was observed between osteoprotegerin and BMD at total body, total hip, and femoral neck^21^. A study by Yano *et al* concluded that the osteoprotegerin serum levels were negatively correlated with bone mineral density at various sites (lumbar spine, femoral neck and total body) and positively correlated with biochemical markers of bone turnover^17^.

Receiver operator curve analysis was done based on the direction of assessment. Osteoprotegerin (u/ml) was evaluated for prediction of cut-off values between control and cases. The results suggested a cut-off value of ≥10.5 u/ml (86.7% sensitive and 80% specific with accuracy of 84.5%) between normal and osteoporosis. Youssef *et al* found osteoprotegerin to be the most diagnostic bone marker to discriminate females with reduced bone mineral density from normal subjects. As regards high validity and overall accuracy, area under curve was 0.871, at cut off value ≤10.9 u/ml; serum osteoprotegerin showed 93.75% sensitivity and 91.7% specificity. The main limitations of the current study were small size of the studied sample, single centric study, and short period of the study and lacunae of literatures on osteoporosis in Indian patients using serum osteoprotegerin as a diagnostic indicator.

## Conclusion

Osteoprotegerin is a valid biomarker to diagnose postmenopausal women with low bone mineral density. The results suggested a cut-off value of ≥10.5 u/ml (86.7% sensitive and 80% specific with accuracy of 84.5%) between normal and osteoporosis. This may suggest a new promising measure to early diagnose patients at high risk of low bone mineral density and subsequently giving early appropriate treatment.

## Acknowledgement

The authors acknowledge the support of the Department of Biochemistry, Era’s Lucknow Medical College in conducting the study.

## Conflict of Interest

There was no conflict of interest in this study and no external funding was received.
